# Mediating effect of social interaction anxiety between emotional intelligence and life satisfaction in physical education students: post-COVID-19 study

**DOI:** 10.3389/fpsyg.2023.1284664

**Published:** 2023-10-02

**Authors:** Juan José Calleja-Núñez, Antonio Granero-Gallegos, Roberto Espinoza-Gutiérrez, Raúl Baños

**Affiliations:** ^1^Faculty of Sports, Autonomous University of Baja California, Tijuana, Mexico; ^2^Department of Education, University of Almeria, Almeria, Spain; ^3^Health Research Centre, University of Almeria, Almeria, Spain; ^4^Department of Musical, Plastic and Corporal Expression, Faculty of Education Sciences, University of Granada, Ceuta, Spain

**Keywords:** social anxiety, emotional repair, emotional clarity, emotional attention, university

## Abstract

The aim of this research is to analyze the effect of social interaction anxiety on satisfaction with life mediated by emotional intelligence. The research design was descriptive, cross-sectional, and non-randomized. In total, 1,164 Mexican physical education students participated (*M*_age_ = 21.21; *SD* = 3.26; 30.0% female; 69.6% male; 0.4% other). The scales used were the Social Interaction Anxiety Scale, Trait Meta-Mood Scale and Satisfaction with Life and a structural equation analysis with latent variables was conducted. The results highlight that it can be asserted that emotional clarity and repair had a mediating effect between social interaction anxiety and satisfaction with life, as they did decrease the negative effects of social interaction anxiety on satisfaction with life. In addition, social interaction anxiety had a direct and positive effect on emotional attention and a negative effect on emotional clarity and repair.

## 1. Introduction

The restrictive measures in response to the COVID-19 pandemic triggered serious consequences on the general health of the world’s population (Faraci et al., 2022). The spread of the pandemic and the ensuing restrictive policies involving confinement, social distancing and mobility had a major impact on the global economy and the daily lives of people around the world (Di Crosta et al., 2020; Xiong et al., 2020; Gollwitzer et al., 2021; Kuo et al., 2021; Racine et al., 2022). These strategies came with severe negative psychological effects (Özdin and Bayrak Özdin, 2020; Pietrabissa and Simpson, 2020; Obschonka et al., 2021), including anxiety, depression, fear of illness, fear of death, fear of social interaction, post-traumatic stress and insomnia (Di Crosta et al., 2020; Rodríguez-Hidalgo et al., 2020; Torales et al., 2020; Sturgill et al., 2021). Said restrictive measures, meant to decrease community transmission (Weeden and Cornwell, 2020), even led to universities shutting down worldwide (Baños et al., 2022).

As a consequence of the COVID-19 pandemic, university education had to undergo a rapid transition from in-person classes to online learning systems (Baños et al., 2021). This increased the daily frequency of technology overuse among university students, especially smartphones (Bhatnagar et al., 2021). This in turn amplified the irrational fear and stress experienced by middle school (Nguyen et al., 2022) and university students (Zwilling, 2022) of being away from a device that facilitates both general communication and attending academic activities, especially in students who struggle to regulate their emotions (Ercengiz et al., 2020; Brown and Medcalf-Bell, 2022). Thus, COVID-19 not only comes with a dreadful pathology, but it is also the source of numerous secondary problems, such as becoming addicted to the use of the Internet, social networks and any form of media associated with recent technologies (Masrek et al., 2022). In fact, the disruptive use of smartphones surged from the onset of the first wave of COVID-19 (Zwilling, 2022), thereby increasing the incidence of pathologies such as stress and anxiety (Nguyen et al., 2022), which even doubled in some countries during the first confinement period (Amerio et al., 2021; Medda et al., 2022). Therefore, as the amount of time spent on screens (e.g., smartphones, computers, tablets, etc.) increases, physical interaction among people diminishes, which has an impact on their social interaction skills (Masrek et al., 2022). For all these reasons, it would be interesting to determine if the levels of social interaction anxiety (SIA) among students can affect their satisfaction with life once the confinement and social distancing measures are lifted and on-site classes return in a post-pandemic context. Despite the significance that the students’ emotional regulation can have in this situation, few studies have analyzed the effect of SIA on satisfaction with life taking into account the emotional intelligence of the students once they have returned to in-person classes at universities following the end of confinement.

### 1.1. Social interaction anxiety

SIA refers to intense, individual emotional reactions and avoidance behaviors, such as fear, anxiety and distress regarding one or multiple social interactions (Li, 2020). SIA is a widespread condition that can sometimes become chronic, causing severe impact on a person’s academic, occupational and social functioning, as well as their psychological well-being on a general level (Wittchen et al., 2000; Kessler, 2003; O’Toole et al., 2013). The fear of being judged or negatively criticized is the core motive that, together with the fear of contracting COVID-19, prompted people to avoid social interactions, which in turn affected the individuals’ daily functioning (Erliksson et al., 2020). Not addressing this pathology immediately and otherwise allowing it to develop in young people may lead to detrimental effects on their mental health and undermine their academic work and their lives in general (Chartrand et al., 2011). As previously mentioned, social interaction was rare or even non-existent during the pandemic, which could easily trigger SIA and unhealthy emotions, especially in young students who continued their education at home (Hahn, 2020). As age increases, so do the academic load and pressure put on young people, which can lead to an increase in interpersonal communication problems; therefore, the social environment can become overwhelming, leading to psychological conditions common in contemporary youth (Li, 2020). This can be particularly detrimental to students about to graduate, as their interpersonal environment has grown increasingly complex and they may become anxious more easily when facing interpersonal problems (Kwon et al., 2018).

People with high levels of SIA exhibit low self-esteem, depressive symptoms and increased dissatisfaction with life (Makadi and Koszycki, 2020). SIA has also been related to obsessive-compulsive disorders, depression and generalized anxiety disorder (Erliksson et al., 2020). A major characteristic of people with SIA is a lack of emotional regulation (Kashdan and Steger, 2006; Kashdan and Breen, 2008; Werner et al., 2011). Therefore, acknowledging or understanding emotions may play a significant role in the adaptive regulation of emotions during social interactions that cause the person to become anxious (O’Toole et al., 2013).

### 1.2. Emotional intelligence

In the university context, emotional intelligence has been highlighted as an adequate tool for coping with stressful situations and achieving successful academic performance and emotional well-being (Parhiala et al., 2018; Guil et al., 2021). Emotional intelligence is defined as an individual’s ability to assess and regulate their own emotions and use them to solve problems and accomplish goals (Salovey et al., 1995). Guil et al. (2021) propose that emotional intelligence is composed of three dimensions: emotional attention (i.e., self-perceptions about the degree to which an individual addresses their own emotional experiences), emotional clarity (i.e., self-perceptions regarding how clearly people understand emotional states) and emotional repair (i.e., self-perceptions about the ability to adequately manage emotions). In general, research has found that higher scores on emotional intelligence are associated with better psychological functioning and well-being, whereas low scores are linked to anxiety (Berenbaum et al., 2003; García-Fernández et al., 2015). Likewise, there are individual differences in the degree of the three dimensions (attention, clarity and repair), with each dimension having a different role (García-Fernández et al., 2015). Enhancing our understanding of broad emotional constructs and discrete emotions in SAD can have implications for theoretical models of SAD, for clinical assessment and diagnosis, and for treatment (Rozen and Aderka, 2023). Numerous authors have suggested further research on how these three dimensions interact and their relationship with SIA (Turk et al., 2005; Boden and Berenbaum, 2012; García-Fernández et al., 2015; Guil et al., 2019).

In this vein, the predisposition of SIA increases when one does not pay attention to the information that emotions provide or possesses high emotional attention but poor emotional clarity (Boden and Berenbaum, 2012). The role of emotional attention is less clear than those of emotional repair and clarity (García-Fernández et al., 2015). On the one hand, although individuals must pay at least some attention to their emotions in order to understand them and to remediate negative ones, high levels of attention have been found to be detrimental to emotional well-being (Salovey et al., 1995). On the other hand, emotional attention has been negatively related to SIA (Turk et al., 2005; Guil et al., 2019). As can be seen, there is controversy regarding the relationship between SIA and the dimension of emotional attention.

Because the ability to clearly identify one’s emotions is the first step to successful emotional regulation and coping (Butler et al., 2006), the importance of emotional clarity in regulating emotions has been particularly underscored. In this case, as opposed to the dimension of emotional attention, most studies agree that a lack of emotional clarity greatly increases SIA (Dixon-Gordon et al., 2014; Thompson et al., 2017; Butler et al., 2018; Guil et al., 2019). Likewise, individuals with lower levels of emotional clarity tend to describe more paranoid beliefs (Boden and Berenbaum, 2012). O’Toole et al. (2013) suggest that deficits in emotional clarity and difficulties in remediating negative emotions are key factors to consider when addressing SIA. In fact, several studies have negatively related emotional repair to SIA (Bigman et al., 2015; Klemanski et al., 2017; Guil et al., 2019; Masters et al., 2019). Specifically in the university context, it has been found that students who hoped to be more successful in regulating their negative emotions showed fewer signs of anxiety (Catanzaro and Mearns, 1999). Furthermore, middle school students who exhibited higher levels of emotional intelligence during the pandemic scored higher on satisfaction with life (Correa-Barwick et al., 2022; Torres-Gázquez et al., 2023). Along these lines, Sturgill et al. (2021) found that a Mindfulness program with university students increased their emotional intelligence and satisfaction with life, however, studies on this population conducted during the pandemic are scarce.

### 1.3. Psychological well-being

The concept of psychological well-being is closely linked to the subjective well-being and the quality of life or satisfaction with life (SWL) concepts (Baños et al., 2019). Diener and Emmons (1985) postulated the Subjective Well-Being Theory to analyze people’s SWL, defining “subjective well-being” as the subjective assessment of one’s own life quality, that is, the range of elements from transitory stages to relatively abstract assessments or evaluations of the meaning of one’s life. These authors stated that people can express being satisfied with their lives either from a global evaluation or after making different assessments in specific areas of their lives (e.g., family, work, social relationships, etc.; Diener and Emmons, 1985). Several studies have associated SWL with low levels of SIA (Wittchen et al., 2000; Kessler, 2003; O’Toole et al., 2013) and with high levels of emotional intelligence (Hodzic et al., 2016; Sánchez-Álvarez et al., 2016; Blasco-Belled et al., 2020).

In terms of the dimensions of emotional intelligence, a study conducted in Spain, Portugal and Brazil found that SWL was predicted by emotional clarity and emotional repair, but not by emotional attention (Hodzic et al., 2016). Blasco-Belled et al. (2020) also found that emotional attention was negatively related to subjective well-being, albeit this relationship was not significant in the study conducted by Ramos-Díaz et al. (2019). In this line, it is emphasized that a decrease in emotional attention and an increase in emotional clarity and repair should be the target of interventions in adolescents to improve their SWL (Guerra-Bustamante et al., 2019; Martínez-Marín and Martínez, 2019; Azpiazu et al., 2022; De la Barrera et al., 2023), since the intelligent management of emotions helps to prevent negative feelings and fosters positive ones, thus promoting greater SWL (Sánchez-Álvarez et al., 2016). Several studies have highlighted the importance of emotional clarity and repair for improved psychological functioning (Petrides et al., 2018; Masters et al., 2019), psychological adjustment (Salguero et al., 2012; García-Fernández et al., 2015; Butler et al., 2018) and psychological well-being (Gohm and Clore, 2002; Extremera and Fernández, 2005; Salguero et al., 2012).

### 1.4. The present study

**Figure 1:**
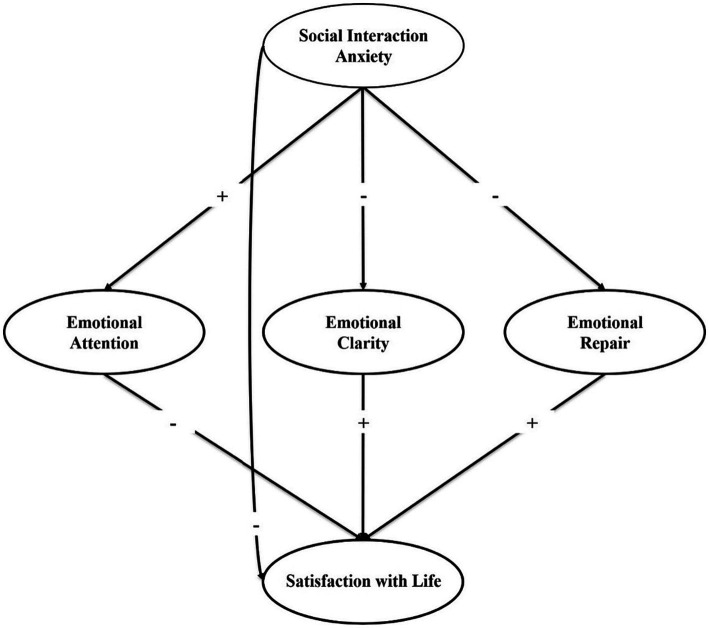
Hypothesized model with the expected correlations.

After analyzing the scientific literature and observing the importance of emotional clarity and repair in preventing SIA and improving people’s SWL, both before and during the COVID-19 pandemic, the predictive analysis of SIA and emotional intelligence on SWL can be deemed relevant, understood that the sanitary restrictions have been lifted. In summary, on the one hand, studies have related SIA to emotional intelligence (Parhiala et al., 2018; Guil et al., 2021) and SIA to satisfaction with life (Wittchen et al., 2000; Kessler, 2003; O’Toole et al., 2013), and, on the other hand, emotional intelligence to satisfaction with life (Hodzic et al., 2016; Sánchez-Álvarez et al., 2016; Blasco-Belled et al., 2020). All these studies were conducted before or during the pandemic, however, we are unaware of the existence of studies that have analyzed emotional intelligence as a mediating variable between SIA and SWL, and if the relationships between these variables have been analyzed following the end of mobility restrictions and the return to in-person university classes. Moreover, research conducted with Mexican students was scarce even before the pandemic. Therefore, this study represents a contribution to the understanding of the relationships among SIA, the dimensions of emotional intelligence and SWL in the Mexican university context. Thus, the objective of this research is to analyze the effect of SIA on SWL mediated by emotional intelligence. Figure 1 shows the hypothesized model for examining the relationships described above. The Strengthening the Reporting of Observational Studies in Epidemiology (STROBE) initiative (Von Elm et al., 2008).

## 2. Materials and methods

### 2.1. Design and participants

The research design was descriptive, cross-sectional, observational and non-randomized. The sample was composed of students from the Faculty of Sport of the three campuses (Campus Ensenada, Campus Mexicali and Campus Tijuana) of the Autonomous University of Baja California (Mexico). Inclusion criteria were the following: (i) to be enrolled in the Bachelor’s degree in Physical Activity and Sport at the aforementioned campus and university; exclusion criteria: (i) failure to provide their informed consent for data use in the study; (ii) failure to duly fill out the data collection form. An *a priori* analysis of the necessary sampling size was conducted to provide an answer to the study objective, considering a structural equation model (SEM) composed of five latent variables and 53 observable variables. The analysis was conducted using the *Free Statistics Calculator* v.4.0 software (Soper, 2023) and a minimum of 1,151 participants was calculated to detect effect sizes (f^2^) = 0.163, with a statistical power of 0.99% and a significance level of α = 0.05. In this research 1,164 physical education students (30.0% women; 69.6% men; 0.4% other) from the three campuses of the Faculty of Sports of the Autonomous University of Baja California (19.8%, Campus Ensenada; 30.7%, Campus Tijuana; 49.6%, Campus Tijuana) participated, aged between 17 and 50 years old (*M* = 21.21; *SD* = 3.26). There were no lost values in the responses included in the study. Apart from the total sample, 29 questionnaires were discarded because they were filled incorrectly, and 14 because the respondents did not give their consent to participate in the research.

### 2.2. Instruments

#### 2.2.1. Interaction Anxiety Scale

This study used the scale adapted to the Mexican context by de la Rubia et al. (2013) based on the original version by Mattick and Clarke (1998). This instrument is composed of 20 items that measure social interaction anxiety (e.g., I find it hard to socialize with my classmates. “*Se me hace difícil socializar con las personas con las que estudio*”). Answers were collected using a 5-point Likert scale ranging from 0 (*not at all*) to 4 (*completely*). For this study, the CFA (Confirmatory Factorial Analysis) goodness-of-fit indices were acceptable: *χ*^2^/df = 5.01, *p* < 0.001; CFI = 0.98; TLI = 0.98; RMSEA = 0.059 (90%CI = 0.047, 0.070; *p*_close_ = 0.077), SRMR = 0.033.

#### 2.2.2. Emotional intelligence

This study used the Mexican version by Valdivia et al. (2015) adapted from the original version by Salovey et al. (1995). The scale contains 28 items that measure emotional intelligence across three dimensions: *emotional attention* (8 items; e.g., I frequently think about my feelings. “*A menudo pienso en mis sentimientos*”), *emotional clarity* (8 items; e.g., I am capable of understanding my feelings. “*Puedo llegar a comprender mis sentimientos*”), and *emotional repair* (8 items; e.g., When I feel sad, I think about all the pleasures in life. “*Cuando estoy triste, pienso en todos los placeres de la vida*”). Answers were collected using a Likert scale ranging from 1 (*completely disagree*) to 5 (*completely agree*). For this study, the CFA goodness-of-fit indices were acceptable: *χ*^2^/gl = 4.97, *p* < 0.001; CFI = 0.98; TLI = 0.97; RMSEA = 0.058 (90%CI = 0.046, 0.068; *p*_close_ = 0.072), SRMR = 0.042.

#### 2.2.3. Satisfaction with life

This study used the scale adapted into Spanish by Atienza et al. (2000) to measure satisfaction with life in general based on the original version by Diener and Emmons (1985). The scale contains 5 grouped items that measure satisfaction with life (e.g., My life circumstances are good. “*Las circunstancias de mi vida son buenas*”). Answers were collected using a Likert scale ranging from 1 (*completely disagree*) to 5 (*completely agree*). For this study, the CFA goodness-of-fit indices were acceptable: *χ*^2^/gl = 2.55, *p* = 0.054; CFI = 0.99; TLI = 0.99; RMSEA = 0.037 (90%CI = 0.000, 0.070; *p*_close_ = 0.071), SRMR = 0.011.

### 2.3. Procedure

First, a meeting was held with the three deputy directors and the general director of the Faculty of Sports of the three campuses of the Autonomous University of Baja California (Ensenada, Mexicali and Tijuana). The purpose of the study was explained and permission to apply the questionnaires was requested. Upon granted authorization, the participants were summoned to the institution’s computer room in March 2022. Participants were taught how to fill out the online questionnaires and informed about the importance of the research, that their participation was anonymous, and that there were no right or wrong answers; they were thus asked to be completely honest and were told that they could abandon the study at any time if they desired so. The questionnaire was completed in around 20 min and all participants gave their prior consent for their responses to be included in the study. The research protocol was approved by the Bioethics Committee of the University of Almeria (Ref: UALBIO2023/001).

### 2.4. Statistical analysis

A structural equation model (SEM) with latent variables was carried out to analyze how SIA is associated with emotional intelligence and satisfaction with life in Mexican university students. For the SEM, a two-step method following Kline (2016) was developed. In step-1, bidirectional relationships between variables were evaluated (i.e., measurement model). In step-2, the predictive effects between the variables were assessed. The SEM was controlled by the variable sex and campus of origin. The following indices were used to evaluate the models: chi square/degrees of freedom (χ^2^/df), CFI (Comparative Fit Index), TLI (Tucker–Lewis Index), RMSEA (Root Mean Square Error of Approximation) with a confidence interval of 90% (CI), and SRMR (Standardized Root Mean Square Residual). For the *χ*^2^/gl ratio, values <2.0 or <5.0 are, respectively, considered excellent (Tabachnick and Fidell, 2019) or acceptable (Hu and Bentler, 1999); for the CFI and TLI, values >0.95 are considered excellent, whereas the range between 0.90 and 0.95 is considered acceptable; for the RMSEA and SRME, values <0.06 are considered excellent, and <0.08, acceptable (Hu and Bentler, 1999; Marsh et al., 2004) Due to the lack of multivariate normality in the SEM (Mardia’s coefficient = 106.82; *p* < 0.001) the maximum likelihood (ML) method was used with the *bootstrapping* procedure for 5,000 re-samplings (Kline, 2016). The reliability of each scale was assessed using different parameters: McDonald’s omega (ω), composite reliability (CR), and AVE for measuring convergent validity. Reliability values >0.70 and AVE > 0.50 are deemed acceptable. For this study, even if the SWL scale yields an AVE value <0.50 (i.e., 0.47), such value is deemed acceptable according to Hair et al. (2018), as all the standardized regression weights were significant and >0.50.

## 3. Results

### 3.1. Preliminary results

Descriptive statistics and correlations between the different variables are shown in Table 1.

**Table 1:** Descriptive statistics and correlations among variables.

Variable	Range	M	SD	Q1	Q2	ɷ	CR	AVE	2	3	4	5
Emotional attention	1–5	3.47	1.10	−0.35	−0.65	0.89	0.89	0.67	0.21^**^	0.08^**^	0.08^**^	0.05
Emotional clarity	1–5	3.43	1.05	−0.30	−0.58	0.90	0.82	0.60	-	0.33^**^	−0.27^**^	0.37^**^
Emotional repair	1–5	3.76	1.03	−0.64	−0.26	0.85	0.87	0.69		-	−0.16^**^	0.37^**^
Social interaction anxiety	0–4	1.47	1.05	0.39	−0.76	0.93	0.90	0.54			-	−0.29^**^
Satisfaction with life	1–5	3.67	0.84	−0.44	−0.26	0.81	0.81	0.47				-

M = mean; SD = standard deviation; Q1 = skewness; Q2 = kurtosis; ɷ = omega of McDonald; CR = composite reliability; AVE = average variance extracted.

* Correlation is significant at level 0.05. ^**^Correlation is significant at level 0.01.

### 3.2. Main results

**Figure 2:**
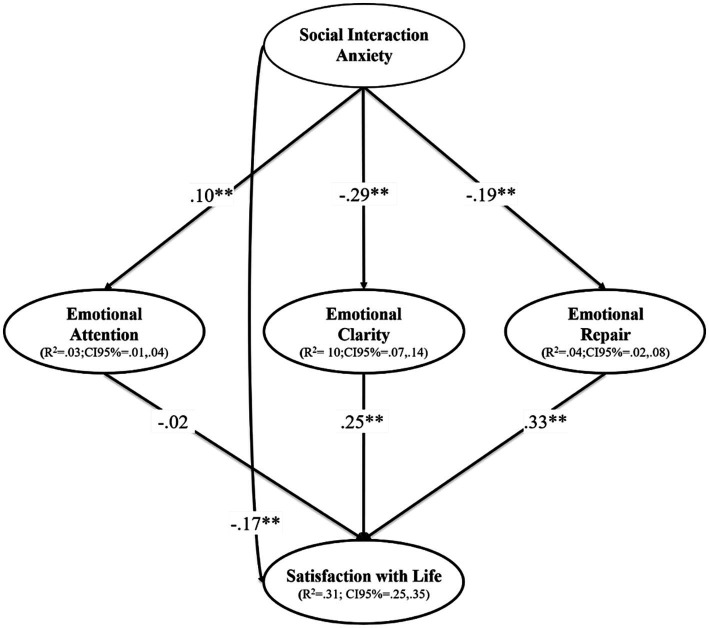
Predictive relationships of the emotional intelligence on satisfaction with life through the mediating role of the social interaction anxiety. **p < 0.01; *p < 0.05. R^2^ = Explained variance; CI = Confidence interval. The dashed lines represent non-significant relationships. The model was controlled by the sex and the campus of the students.

During step 1, the SEM showed excellent goodness-of-fit indices: *χ*^2^/gl = 2.40, *p* < 0.001; CFI = 0.98; TLI = 0.97; RMSEA = 0.035(90%CI = 0.031; 0.038; *p*_close_ = 1.00), SRMR = 0.035. During step 2, the hypothesized SEM yielded a similar and excellent fit: *χ*^2^/gl = 2.40, *p* < 0.001; CFI = 0.98; TLI = 0.97; RMSEA = 0.035(90%CI = 0.031; 0.038; *p*_close_ = 1.000), SRMR = 0.035. The model was controlled by the sex and campus of origin variable and reached an explained variance of 31% for SWL, 3% for emotional attention, 4% for emotional repair and 10% for emotional clarity (Figure 2). The correlations among SIA, the emotional intelligence variables (i.e., emotional attention, emotional clarity, and emotional repair) and SWL can be attested in Figure 2 and Table 2.

Figure 2 outlines the SEM and shows that SIA has a direct, negative and significant relationship with SWL (*p* < 0.001), as well as emotional clarity (*p* < 0.00) and emotional repair (*p* < 0.001). On the other hand, SIA has a direct, positive and significant relationship with emotional attention (*p* = 0.004). In terms of the mediating effect of the dimensions of emotional intelligence, it should be noted that emotional clarity and emotional repair play a remarkable role between SIA and SWL, as they soften the negative direct effect of SIA on SWL, given that these two dimensions (i.e., emotional clarity and emotional repair) have a positive and significant direct effect on SWL (Table 2). In addition, Figure 2 demonstrates the CI (95%) of R^2^, thereby confirming that these values can be considered ES measurements (Dominguez-Lara, 2017).

**Table 2:** Estimation of significant standardized parameters and statistics of the mediation model.

	Independent variable	Dependent variable	Mediator	β	SE	95%CI	
						Inf	Sup
Direct effects							
	SIA	Emotional attention		0.10^**^	0.04	0.03	0.16
	SIA	Emotional clarity		−0.29^**^	0.04	−0.35	−0.23
	SIA	Emotional repair		−0.19^**^	0.03	−0.24	−0.13
	SIA	SWL		−0.17^**^	0.04	−0.22	−0.11
	Emotional clarity	SWL		0.25^**^	0.05	0.17	0.33
	Emotional repair	SWL		0.33^**^	0.04	0.25	0.40
Indirect effects							
	SIA	SWL	Emotional Clarity	−0.07^**^	0.01	−0.09	−0.04
	SIA	SWL	Emotional Repair	−0.06^**^	0.01	−0.07	−0.03
Total effects							
	SIA	SWL		−0.30^**^	0.03	−0.36	−0.24

β = Estimation of standardized parameters; SE = standard error; 95% CI = 95% confidence interval; Inf = Inferior limit of 95% CI; Sup = Superior limit of 95% CI. SIA = social interaction anxiety; SWL = satisfaction with life.

* *p* < 0.05. ^**^p < 0.01.

## 4. Discussion

The purpose of this research was to analyze emotional intelligence as a mediator between SIA and SWL. The main results illustrate the important role of emotional clarity and repair as mediating variables between SIA and SWL, given that they decrease the negative effect of SIA on SWL.

A possible explanation for this is that emotional clarity is the key to regulating emotions, as the ability to clearly identify one’s emotions is the first step to successful emotional regulation and coping (Butler et al., 2006). Thus, young people who understand their emotions and recognize their own abilities to solve problems and overcome difficult situations through their own efforts will adopt a positive emotional coping style, mitigating the distress caused by SIA and improving their social performance (Li, 2020), and thus their SWL (Hodzic et al., 2016). In this vein, Guil et al. (2019) highlight the importance of the interaction between the three dimensions of emotional intelligence concerning SIA. These authors state that students who are confident in their own abilities to cope with challenging situations, do not pay much attention to their emotions, and do trust their competencies to clearly perceive and repair their emotional states will cope more efficiently with SIA (Guil et al., 2019). In terms of the relationship of SIA with the dimensions of emotional intelligence, we consider that, because of its timely execution, this study provides the global university context with an important scientific contribution, understanding that the data for the present study were collected just 2 weeks after the return to in-person classes following the COVID-19 confinement, as the fear of contagion and stress in general due to the pandemic were still visible in society (Di Crosta et al., 2020; Rodríguez-Hidalgo et al., 2020; Torales et al., 2020).

However, according to our research, emotional attention did not have a significant direct effect on SWL, nor were there significant indirect effects between SIA and SWL. In this line, Hodzic et al. (2016) also did not obtain a significant relationship between emotional attention and SWL. Although the role of emotional attention compared to emotional clarity and repair is confusing according to García-Fernández et al. (2015), on the one hand, Blasco-Belled et al. (2020) have found that emotional attention negatively and significantly predicts SWL (Blasco-Belled et al., 2020), while others found a negative but not significant prediction (Hodzic et al., 2016; Ramos-Díaz et al., 2019). Emotional attention not predicting SWL could be due to the fact that this dimension does not have as much of a potential inference in people’s behavior as emotional clarity and emotional repair do (Salovey et al., 1995). Thus, when students pay too much attention to their emotions without understanding them or having repair skills, they negatively affect their mood and psychological functioning (García-Fernández et al., 2015; Butler et al., 2018; Petrides et al., 2018; Masters et al., 2019), while also decreasing their SWL (Guerra-Bustamante et al., 2019; Martínez-Marín and Martínez, 2019; Azpiazu et al., 2022; De la Barrera et al., 2023). On the contrary, the intelligent management of emotions helps students to prevent negative feelings and increases positive ones, contributing to the increase of SWL (Sánchez-Álvarez et al., 2016) while improving academic performance with appropriate learning strategies (García-Fernández et al., 2015). Because scientific literature in this context is scarce, and given that the results obtained are in line with the international literature, we consider this study to be a scientific contribution to the Mexican university context.

It is also worth mentioning that SIA significantly, positively and directly predicted emotional attention in this research. Since past studies have negatively related SIA to emotional attention (Turk et al., 2005; Guil et al., 2019), contrary to the results obtained in this research, the relationship between SIA and emotional attention remains controversial (García-Fernández et al., 2015). However, the results obtained in the present study are in line with the findings of Boden and Berenbaum (2012), who also described a positive relationship between SIA and emotional attention. These authors claim that when one does not pay attention to the information provided by emotions, or one has a high level of emotional attention, but with a deficient emotional clarity, the predisposition toward SIA increases. A potential explanation is that people with a high level of emotional attention tend to be hypervigilant about their own emotions and signs of anxiety, becoming less flexible to explain their states of anxiety and misjudging both their severity and visibility to others, and feeling more threatened by how others understand them (Roth et al., 2001; Wells and Papageorgiou, 2001; Edelmann and Baker, 2002).

Finally, we will describe a series of limitations and strengths of the present study, as well as future research perspectives. Limitations include: (i) the timing of data collection, 2 weeks after the confinement and mobility measures due to the COVID-19 pandemic had been lifted, as this might have caused widespread emotional and psychological instability in participants, even when filling the questionnaires; (ii) the variables were evaluated neither before nor during the pandemic, so we are not able to observe the evolution of these variables after such an upsetting experience; (iii) the cross-sectional design of the study does not allow for establishing causal inferences; (iv) there was no sample randomization, so the results cannot be generalized; (v) a possible social desirability bias due to the use of self-reporting, since participants may have exaggerated their responses. On the other hand, noteworthy strengths of this research include: (i) the timing of data collection may be a strength in itself since scientific literature on this topic set just after the end of mobility restrictions is scarce; (ii) the sample size of Mexican undergraduate Physical Education students from the three campuses of the Autonomous University of Baja California (Ensenada, Tijuana and Mexicali), as well as the statistical power of the study. We consider it necessary for future studies to analyze the dimension of emotional attention by conducting a quadratic regression analysis since both excessive emotional attention and low levels of this dimension are related to SIA. We also suggest longitudinal studies a few years after the pandemic to analyze how the post-pandemic dimensions of emotional intelligence relate to occupational success.

## 5. Conclusion

In conclusion, it can be asserted that emotional clarity and repair had a mediating effect between SIA and SWL, as they did decrease the negative effects of SIA on SWL. In addition, SIA had a direct and positive effect on emotional attention and a negative effect on emotional clarity and repair. Furthermore, emotional clarity and repair had a direct and positive effect on SWL, although emotional attention did not predict SWL. Therefore, we believe that university institutions should train and educate students in managing SIA by further developing their emotional intelligence. It would be interesting to provide university students with strategies to control and manage the understanding of their own emotions, and to manage negative emotions resulting from SIA. Finally, it is important to highlight the importance of young people not paying too much emotional attention, as an excess of it can increase SIA levels, undermining SWL at the same time.

## 6. Practical implications

The results of this research underline the importance of emotional clarity and repair in decreasing SIA and increasing SWL in Mexican university students after the pandemic and are in line with other studies conducted before the pandemic. Therefore, educational institutions should organize workshops related to the development of emotional intelligence to help young people to understand the feelings and emotions that they experience, and to remediate negative emotions that might be stressful for them (Correa-Barwick et al., 2022; Cabello-Sanz and Muñoz-Parreño, 2023; Torres-Gázquez et al., 2023). In this line, Valenti et al. (2022) consider that appropriate programs should be designed to help people to see the bright side of negative experiences, which permits a reshaping of harmful emotional outcomes by focusing on some positive aspects. In addition, it is recommended that people suffering from SIA practice activities such as Mindfulness (Butler et al., 2018), aerobic exercise (Jazaieri et al., 2012), or activities in natural environments (Chen and Huo, 2022), as they help to decrease SIA and to increase the levels of SWL.

## Data availability statement

The raw data supporting the conclusions of this article will be made available by the authors, without undue reservation.

## Ethics statement

The studies involving human participants were reviewed and approved by the University of Almería (Ref: UALBIO2023/001). The patients/participants provided their written informed consent to participate in this study.

## Author contributions

JC-N: Conceptualization, Data curation, Formal analysis, Writing – review & editing. AG-G: Conceptualization, Formal analysis, Methodology, Writing – review & editing. RE-G: Data curation, Investigation, Writing – review & editing. RB: Conceptualization, Data curation, Formal analysis, Methodology, Writing – original draft.
